# Properties of Flicker ERGs in Rat Models with Retinal Degeneration

**DOI:** 10.5402/2012/346297

**Published:** 2012-05-22

**Authors:** Jing An, Qun Guo, Li Li, Zuoming Zhang

**Affiliations:** Department of Clinical Aerospace Medicine, Fourth Military Medical University, Xi'an, China

## Abstract

*Purpose*. To describe the characteristics of rod and cone functions in rat models for congenital stationary night blindness (CSNB) and retinal cone dysfunction (RCD). *Methods*. Rod and cone function were isolated by recording the rod-/cone-driven flicker and blue light flicker electroretinograms (ERGs). *Results*. During dark adaptation, the amplitudes of flicker ERGs in CSNB rats were lower than those in control rats; the responses of RCD rats were similar to control rats. During light adaptation, the amplitudes of flicker ERGs in CSNB rats were reduced; whereas the responses of RCD rats were not detected. Blue flicker ERGs were not observed in CSNB rats at lower frequencies. The cone driven critical flicker frequencies (CFFs) in control rats were 62 Hz. The rod driven CFF of RCD rats was 20 Hz; whereas the rod-/cone-driven CFF of CSNB rats both were about 25 Hz. *Conclusions*. The function of the rod system was damaged completely, the cones were the source of vision in CSNB rats. Rod system function is excellent in RCD rat. The rods of albinism rats are sensitive to frequencies less than 20 Hz; whereas the cones are sensitive to frequencies up to 62 Hz.

## 1. Introduction

Inherited retinal degeneration disorder is one of the most serious diseases that cause blindness in humans. Gene mutations are expressed in photoreceptors, bipolar cells, or other part of the retina, which can cause various changes in rod and cone function. The loss of rod or cone path function or visual signal transduction results in serious eye disease in humans. However, the pathogenesis of retinal degeneration disorder is still not quite understood, and valid treatment is currently lacking. Clinically, the classification of retinal diseases mainly depends on electroretinograms (ERGs) to analyze the extent of damage to the cone and rod systems.

Spontaneous generation animal models of retinal degeneration are important for studying gene mutation, protein function, and disease prevention, and cure. Congenital stationary night blindness (CSNB) is a nonprogressive retinal degeneration disease characterized by the loss of night vision with partially or completely absent rod function [1]. CSNB disease is transmitted via an autosomal recessive, autosomal dominant or X-linked mode of inheritance. Several groups have shown that the genetic heterogeneity of the X-linked CSNB (XLCSNB) is controlled by two different loci on the X chromosome. The *NYX* gene mutation gives rise to cCSNB (CSNB1), whereas the *CACNA1F* gene mutation leads to iCSNB (CSNB2) [2–7]. Retinal cone dysfunction (RCD) [8] is a retina disease involving the loss of cone vision characterized by a decreased cone function, which always causes achromatopsia or photophobia in the daytime. RCD is especially debilitating to vision in human patients. However, there are few animal models of cone dystrophy [9–11]. Our laboratory has discovered and bred CSNB and RCD rat models to prove X-linked recessive inheritance. We have proved the mutation gene in CSNB rats is localized in 2941 of *Cacna1f*. The mutation leads to the prematurely termination of coding Cav1.4 calcium channel protein and blocked transmission of visual signal in the rod system [7]. The causative gene of RCD rat is *Opn1mw*, which plays a key role in cone system function [12]. These two retinal degeneration strains can become important animal models for studying retinal illness.

Flicker ERG, which is stimulated by a series of frequencies to record continual waves, has always been used in estimating cone and rod system function. Using high-frequency flicker and bright illumination to deactivate rods can be used to determine the function of cone [13]. However, some reports have indicated that the rod system reduces cone flicker sensitivity at high frequencies under dark-adapted condition [14, 15]. Stimulation luminance or adaptation is relative to the intrinsic properties of flicker responses from the rod/cone pathway [16]. The rod-cone interaction causes flicker ERGs. Currently, some studies used pharmacologic manipulation to reduce rod-cone interaction and simulate flicker ERGs in primates. These investigations have proven that light-adapted flicker responses include cone photoreceptors, depolarizing bipolar cells (DBCs), and hyperpolarizing bipolar cells (HBCs) [17, 18]. The activity of the DBC and HBC pathways expressed different characteristics and photoreceptors contributed at low frequencies. Other reports found that the HBCs of nob1 mice, with ERGs similar to the human complete CSNB disease, definitely plays a main role at high frequencies, and DBCs made a relatively small contribution to cone ERGs [19, 20]. Cicerone used the flicker ERG approach to evaluate cone function during the course of progressive degeneration in the Royal College of Surgeons (RCS) rat, which has a primary defect in retinal pigment epithelial cells that leads to the progressive rod death followed by cones damaged [21]. Several studies could characterize retinitis pigmentosa (RP) using flicker responses. A study proved that RP patients have reduced amplitudes with normal response phases [22, 23]. In another report, X-linked retinoschisis (XLRS) patients showed reduced amplitudes at higher temporal frequencies, which indicate ON-bipolar cell contribution to the flicker ERGs [24]. The composition of visual transduction signals can be independently accessed using flicker ERG in more kinds of retinal degenerations.

In our previous research, we had learned the rough extent of damage to vision in the two kinds of retinal degeneration rats [8, 25]. The results indicated that the rod pathway function of the CSNB retina was damaged completely. In addition, we could expel the retinal rod elements to study the survival function of cone system. The cone ERGs in the RCD rats were not elicited [8], which gave us a chance to study rod function. The current research supports future investigations into the interaction of rod and cone systems and provides more evidence of the mechanism of CSNB and RCD diseases.

## 2. Materials and Methods

### 2.1. Breeding of Animal Models

The CSNB and RCD rats were found among Sprague-Dawley (SD) rats checked for their ERG. The CSNB rat has retinal degeneration, which is caused by a *Cacna1f* gene mutation [7]. The RCD rat has the *Opn1mw* gene mutation [12]. Two male mutant rats were mated with age-matched female SD rats (The Fourth Military Medical University, Xi'an, China). The F1 offspring had normal responses in their ERG. The F1 CSNB and RCD female rats were crossed with the original mutation male rats. We then crossed the affected F2 males with the affected F2 females to obtain the two congenital inbred strains. The rats in every generation were identified based on their visual electrophysiology. The F25 CSNB rats and F16 RCD rats were bred.

### 2.2. Animal Preparation

F10 RCD, F20 CSNB, and wildtype (SD) rats were randomly selected. Each group consisted of 6 male rats weighing 180 g to 220 g. The refractor media of all subjects were clear, and the fundi were normal. The RCD and CSNB rats were tested and bred at our barrier animal laboratory with free access to water and food, and maintained on a 12-hour light-dark cycle at a constant temperature of 22°C to 26°C. For comparison, the flicker ERG records of wild-type rats were also obtained. All animal experiments were performed in compliance with the ARVO Statement for the Use of Animals in Ophthalmic and Vision Research and were approved by the Animal Care and Use Committee of the Fourth Military Medical University.

After overnight dark adaptation, the rats were anesthetized intraperitoneally with a compound anesthetic at 0.6 mL/kg body weight. The pupils of the tested rats were dilated with 0.5% tropicamide. Corneal anesthetic (topical proparacaine 0.5%) was given, and the eyes were coated with 1% methylcellulose before a silver chloride wire loop electrode was placed in contact with the cornea. All operations were prepared under dim red light for ERG recordings.

### 2.3. Visual Stimulation and Recording

Stimulations were produced using a full-field stimulation globe with an LED light source positioned 15 cm away from the eye. When the respiration of the rats became steady, the flicker ERGs were recorded using the silver chloride loop electrode applied to the corneal surface. Stainless needle electrodes placed into the cheek and tail served as reference and ground electrodes, respectively. The ERG signals were recorded using a commercial system (RETIport; Roland Consult GmbH, Brandenburg, Germany). Strobe stimulus flashes were delivered in a Ganzfeld.

Flicker recordings were initially obtained after dark adaptation. Scotopic flicker ERGs were elicited in a series of tested temporal frequencies from 1 Hz to 30 Hz with a dim stimulus luminance of −2.5 log*·*cd*·*s/m^2^. Furthermore, rod- and cone-driven critical flicker frequencies (CFFs) were recorded in a stimulus luminance series for the three kinds of rats. Photopic flicker ERGs were accomplished with a background illumination of 25 cd/m^2^ for 10 minutes, and the stimulus frequencies were increased from 1 Hz to 62 Hz with a stimulus luminance of 0 log*·*cd*·*s/m^2^. One week later, we attached a blue filter (wavelength: 480 nm) of the LEDs light (stimulus luminance of −2.5 log*·*cd*·*s/m^2^) and elicited blue scotopic flicker ERG responses in dark adaptation. We used a band pass of 1 Hz to 300 Hz, and 8 replicate responses were averaged for each test. Each stimulus condition was repeated two or three times, and waveforms were recorded for 500 ms.

### 2.4. Analysis

The amplitude and latency of each waveform were measured by using a RETIport (Roland Consult, Germany). The amplitude was determined from the second wave of the flicker response. All data were analyzed statistically using SPSS 11.0 software. Two groups used independent-sample  *t*-test. More than two groups, Levene's statistical analysis was used to test for the homogeneity of variance. If homogeneous variances were found, we used an one-way ANOVA and a Dunnett  *t*-test. Otherwise, we used a Kruskal-Wallis test and a Wilcoxon test. The data are presented as mean ± SE in the figures. The differences were considered significant at *P* < 0.05. Charts were made using Origin 7.0.

## 3. Results

### 3.1. Comparison of the Representative ERGs in the Three Rat Models

**Figure 1:**
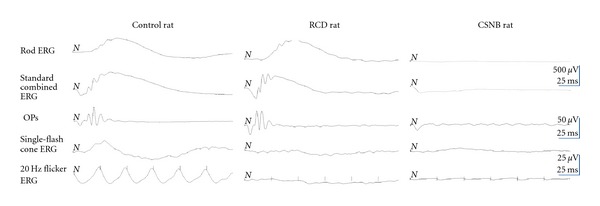
ERG responses of wild-type control (left trace in the panel), RCD (middle trace in the panel), CSNB (right trace in the panel) rats. The rod response was not detected in the CSNB rats; and the amplitude of b-wave cone response and flicker ERGs was markedly reduced in the CSNB rat. The cone response and flicker ERG were not detected in the RCD rats. “ND” means not detected.

The representative ERG responses obtained from the rat models are shown in Figure 1. The amplitudes of the b-wave of the rod ERG responses were not detected in the CSNB rats (Figure 1, right trace in the panel), standard combined ERG showed typical negative waveforms, and the cone response ERGs were markedly reduced in the CSNB rats. A significant delay in the latency of cone response ERGs was observed in the CSNB rats than wild-type rats. However, cone-driven ERGs were not elicited in the RCD rats (Figure 1, middle trace in the panel). The amplitude and latency of the rod ERGs, standard flash ERGs, and oscillatory potentials (OPs) in the RCD rats were similar to that of wild-type rats.

### 3.2. The Properties of Scotopic Flicker ERGs

**Figure 2:**
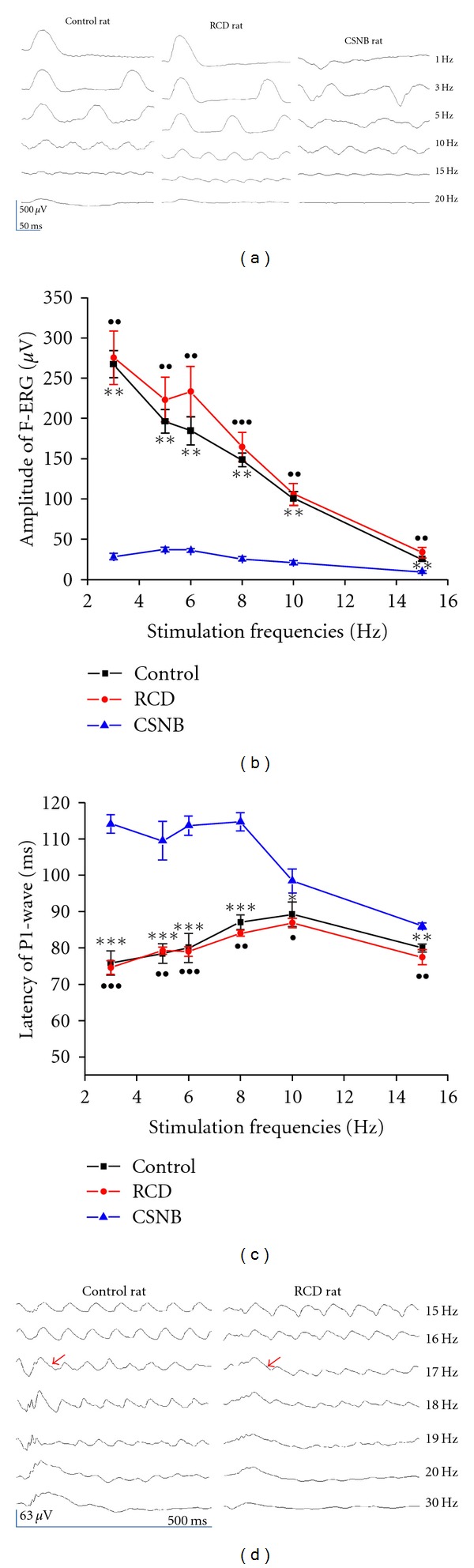
Scotopic flicker responses of three kinds of rats. (a) Representative responses of control (left graph of top), RCD (middle graph of top), and CSNB (right graph of top) rats using a series of temporal frequencies during dark adaptation. Each waveform was recorded for 500 ms, and responses were the averages of ten tests. (b) Amplitudes of the scotopic flicker responses. The amplitudes in the RCD rats were similar to those of the control rats at every frequency (*P* > 0.05). The amplitudes of CSNB rats were significantly lower than that of the control and RCD rats at every frequency. (c) Peak of the scotopic flicker responses. The latency of the flicker responses of the CSNB rats was significant delayed compared with those in the control and RCD rats at every frequency. (d) Characteristics of the scotopic flicker responses from 15 Hz to 30 Hz. The first wave of the flicker response was bigger than continuous waves generally appeared at 17 Hz-18 Hz in both the wild-type control (left panel) and the RCD (right panel) rats. Arrows point to the first bigger wave of flicker response. The amplitudes were determined from the second peak (P_2_) except 1 Hz, and the peak times were determined in the first peak (P_1_) of the flicker responses. The data points indicate the mean (± SE). ***P < 0.001, **P < 0.01, and *P < 0.05 for the wild-type rats versus the CSNB rats ^•••^ *P* < 0.001, ^••^ *P* < 0.01, and ^•^ *P* < 0.05 for the RCD rats versus the CSNB rats. Square: wild-type rat; circle: RCD rat; triangle: CSNB rat.

**Figure 3:**
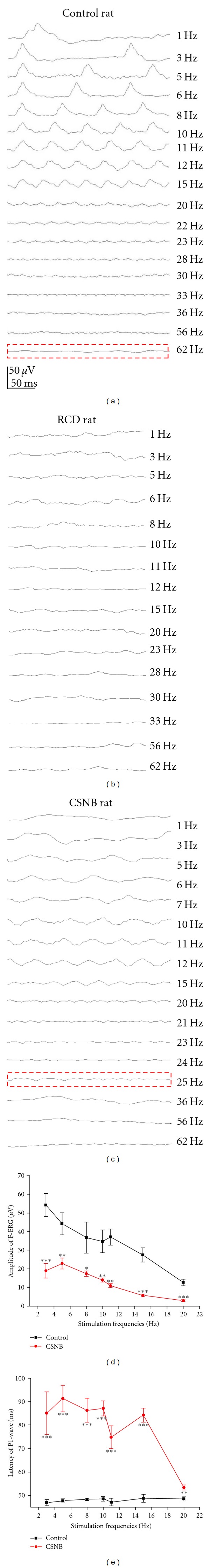
Photopic flicker responses of three kinds of rats. (a) Representative responses of the wild-type control rat at temporal frequencies in light adaptation. (b) Representative responses of RCD rat. (c) Representative responses of CSNB rat. The waveforms from the wild-type rats were detected until 62 Hz; waveforms were not detected at all frequencies in the RCD rats; the flicker responses were undefined at about 23 Hz and not elicited at 25 Hz in the CSNB rats. (d) Amplitudes of photopic flicker responses using a series of temporal frequencies. The amplitudes in the CSNB rats were much lower than that of the wild-type rats at every stimulation frequency. (e) Peak time of photopic flicker responses using a series of temporal frequencies. The peak time of the CSNB rats was significantly delayed compared with that of the wild-type rats. Dashed line noted: flicker responses were not detected at the frequency. The amplitudes were determined from the second peak (P_2_), and peak time was determined from the first peak (P_1_) of the flicker responses. Data points indicate the mean (± SE). ***P < 0.001, **P < 0.01, *P < 0.05 for the wild-type rats versus the CSNB rats. Square: wild-type rat; circle: CSNB rat.

During dark adaptation, the waveforms of the RCD rats were similar to that of the wild-type control rats (Figure 2(a)). The flicker response of the CSNB rats had a negative waveform at 1 Hz, the elicited P_1_ wave under the basic line. However, the P_1_ waves of the control rats and the RCD rats were mainly observed at 1 Hz. At 20 Hz, the flicker responses of the RCD rats were not observable, whereas those of the wild-type rats showed a sine-wave pattern beginning at 30 Hz under scotopic condition (Figure 3(a)).

The amplitudes of the recordings from the CSNB rats was lower than that of wild-type control and RCD rats (*P* < 0.01, Figure 2(b)). The amplitudes of the recordings from the CSNB rats were 1/10, those from the wild-type rats at 3 Hz in flicker responses were 1/7 at 5 Hz, and were 1/4 at 15 Hz The peak time of the CSNB rats was significantly delayed compared with wild-type rats (Figure 2(c)). At increasing frequencies, the differences in amplitude decreased between the CSNB and the wild-type rats. Perhaps the cone system of the CSNB rats was a main component in flicker responses. No significant difference were observed in the amplitudes and peak times between the wild-type and the RCD rats (*P* > 0.05, Figures 2(b) and 2(c)). However, the amplitude of the RCD rats had a small upward inflection point at 6 Hz. And the peak time of RCD rats was delayed compared with the wild-type rats when the frequency exceeded 6 Hz. The first wave of flicker responses was bigger than the subsequent continuous waves, and the first wave included a portion of the second wave. The first bigger wave generally appeared at around 17 Hz or 18 Hz in both the wild-type controls (left panel) and the RCD (right panel) rats (Figure 2(d)).

### 3.3. The Properties of Photopic Flicker ERGs

Under light adaptation, the cone-driven waveforms of the flicker ERGs were not elicited at any frequency in the RCD rats (Figure 3(b)). The photopic flicker waveforms were induced until 62 Hz in the wild-type rats (Figure 3(a)). The photopic flicker responses of CSNB rats were elicited at about 23 Hz and were undetectable at about 25 Hz. Furthermore, high-frequency flicker responses were absent in the CSNB rats in light adaptation (Figure 3(c)).

The amplitudes of photopic flicker responses in the CSNB rats were smaller than those in the wild-type rats in every stimulation frequency (Figure 3(d)). Significant delays in the peak time of photopic flicker responses were observed in CSNB compared with the wild-type rats (Figure 3(e)). The responses of the CSNB rats declined from 5 Hz. At frequencies lower than 10 Hz, the photopic flicker response amplitudes declined progressively with increasing stimulating frequencies in the wild-type rats. At 11 Hz, the wild-type rats displayed the peak amplitude of flicker response, and the peak time was short at 11 Hz.

### 3.4. The CFF Values of the Three Rat Models

**Figure 4:**
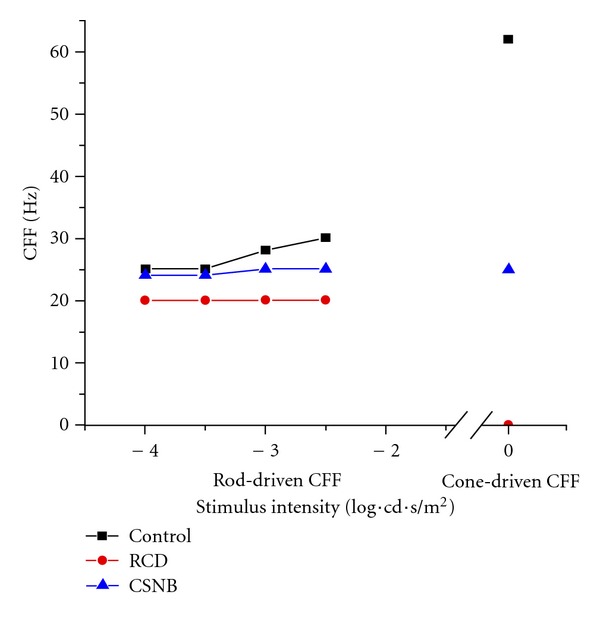
CFF values of the retinal degeneration rats. During dark adaptation using dim light stimulation (−4.0, −3.5, −3.0, and −2.5 log·cd·s/m^2^), the rod-driven CFF of the wild-type control rats increased increasing stimulation. The increase among the CSNB rats was small. The rod-driven CFF among the RCD rats was steady. During light adaptation (background light 25 cd/m^2^) using bright light to stimulate (0 log·cd·s/m^2^), the cone-driven CFF among the wild-type rats was up to 62 Hz. The cone-driven CFF remained unchanged compared with the rod-driven CFF in the CSNB rats (−2.5 log·cd·s/m^2^). The CFF was not detected in the RCD rats. Square: SD rat; circle: RCD rat; triangle: CSNB rat.

The CFF shapes versus intensity curves were obtained from three rat models under different temporal frequencies using different stimulus levels (−4.0, −3.5, −3.0, and −2.5 log·cd·s/m^2^) under dark adaptation, and using 0 log*·*cd*·*s/m^2^ under light adaptation (background light 25 cd/m^2^) (Figure 4). The CFF of the wild-type rats increased with an ascending slope following the increase in intensity. The rod-driven CFF of the wild-type rats grew from 25 Hz to 30 Hz at different stimulus levels under dark adaptation. Under light adaptation, the cone-driven CFF was 62 Hz. The cone-driven CFF was much higher than the rod-driven CFF in the wild-type rats. In contrast, the values for the CSNB rats were not significantly different under dark and light conditions. The rod-driven values for the CSNB rats ranged from 24 Hz to 25 Hz at different stimulus intensities, whereas the cone-driven CFF was 25 Hz. The rod-driven CFF for the RCD rats was 20 Hz under the various stimulus intensities, but cone CFFs were not elicited.

### 3.5. Properties of the Blue Scotopic Flicker ERGs

**Figure 5:**
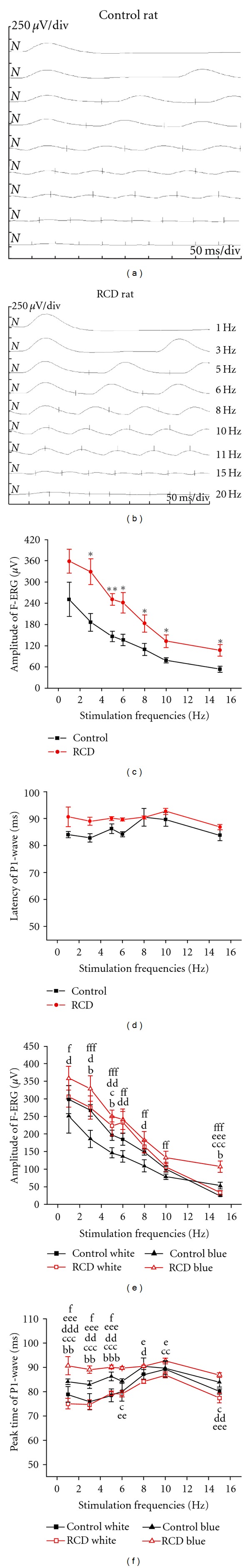
Scotopic flicker responses of retinal degeneration rat models using dim blue light stimulation (−2.5 log·cd·s/m^2^). (a) Flicker responses of the wild-type rat using blue light during dark adaptation. (b) Blue flicker responses of the RCD rat. (c) Amplitudes of the blue flicker responses of the wild-type and the RCD rats. The amplitudes in the RCD rats were significant higher than those in the wild-type rats at every stimulus frequencies. **P < 0.01; *P < 0.05 wild-type rats versus RCD rats. (d) Peak time of the blue flicker responses of the wild-type and RCD rats. Significant differences in latency comparing the wild-type rats with the RCD rats using blue light. Square: control rat. Circle: RCD rat. (e) Amplitudes of the blue and white flicker responses of the wild-type and RCD rats. (f) Peak time of the blue and white flicker responses of the wild-type and RCD rats. ^bbb^ *P* < 0.001, ^bb^ *P* < 0.01, and ^b^ *P* < 0.05 for the wild-type rats using white light stimulation versus wild-type rats using blue light. ^ccc^ *P* < 0.001, ^cc^ *P* < 0.01, and ^c^ *P* < 0.05 for wild-type rats using white light stimulation versus RCD rats using blue light. ^ddd^ *P* < 0.001, ^dd^ *P* < 0.01, and ^d^ *P* < 0.05 for RCD rats using white light stimulation versus wild-type rats using blue light. ^eee^ *P* < 0.001, ^ee^ *P* < 0.01, and ^e^ *P* < 0.05 for RCD rats using white light stimulation versus RCD rats using blue light. ^fff^ *P* < 0.001, ^ff^ *P* < 0.01, and ^f^ *P* < 0.05 for wild-type rats using blue light stimulation versus RCD rats using blue light. Solid square: wild-type rat using white light; hollow square: RCD rat using white light; solid triangle: wild-type rat using blue light; hollow triangle: RCD rat using blue light. The amplitudes were determined using the second peak (P_2_) except at 1 Hz, and the peak times were gotten in first peak (P_1_) of flicker responses. Data points indicate the mean (± SE).

**Figure 6:**
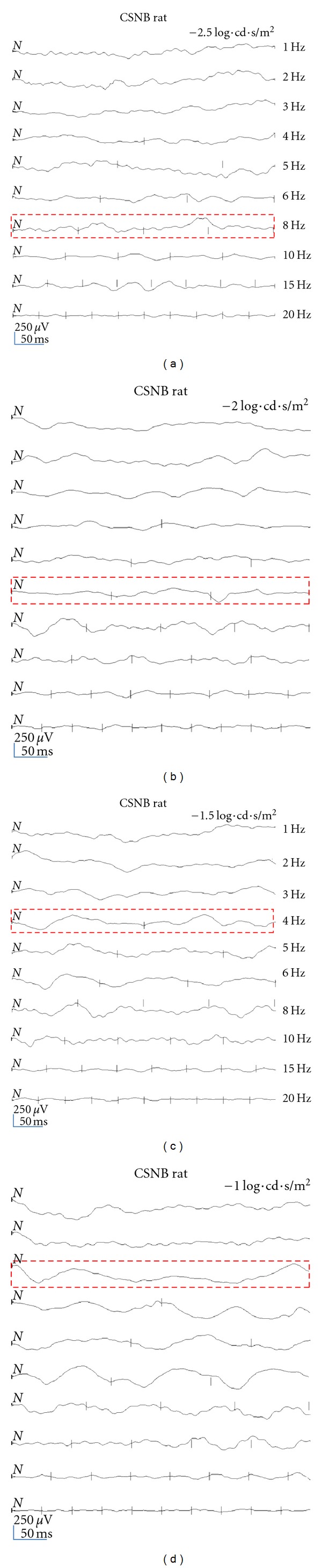
Representative blue flicker responses of a CSNB rat determined using different stimulus intensities and frequencies. Using −2.5, −2.0, −1.5, and −1.0 log·cd·s/m^2^ white LED behind a blue filter to record the flicker ERGs in CSNB rats. (a) No waveforms were detected until above 8 Hz using −2.5 log·cd·s/m^2^ stimulation. (b) Blue flicker responses elicited at 6 Hz using −2.0 log·cd·s/m^2^ stimulation. (c) Blue flicker responses elicited at 4 Hz using −1.5 log·cd·s/m^2^ stimulation. (d) Blue flicker responses elicited at 3 Hz using −1.0 log·cd·s/m^2^ stimulation. Using brighter lights facilitated recording the responses. Dashed lines indicate that blue flicker responses were detected at that frequency in the CSNB rats.

The blue flicker responses of the three groups of rats under scotopic condition are shown in Figure 6. The responses to dim blue light flicker were elicited using white stimulation (−2.5 log·cd·s/m^2^), and a blue filter was placed in front of the tested eyes. Rods are known to be more sensitive to blue light than cones. The amplitudes for the RCD rats were larger than that of the wild-type rats (Figures 5(a), 5(b), and 5(c)). The amplitudes of blue flicker responses were also bigger than the dim white light responses in the RCD rats at every stimulus intensity (Figure 5(e)). The rod function in the RCD rats was normal. The peak times for the RCD rats were shorter than those for the wild-type rats, at 1, 3, 5, 6, and 10 Hz (Figure 5(d)). However, the peak of the blue flicker responses was delayed compared with that of white flicker in both the wild-type and the RCD rats (Figure 5(f)). At 20 Hz, no responses were elicited from the RCD rats during dim blue light stimulation, the same as the result for dim white light stimulation under scotopic conditions. No blue flicker responses were detected until above 8 Hz in the CSNB rats (Figure 6(a)).

We used the brighter light (−2.0, −1.5, and −1.0 log·cd·s/m^2^, white LED, passed through the blue filter, wavelength is 480 nm) to elicit the blue flicker responses from the CSNB rats. No waveforms were detected until above 8 Hz at −2.5 log*·*cd*·*s/m^2^. Flicker waves were elicited at about 6 Hz at −2.0 log·cd·s/m^2^, at about 4 Hz at −1.5 log·cd·s/m^2^, and at about 3 Hz in −1.0 log·cd·s/m^2^ luminance (Figures 6(a), 6(b), 6(c), and 6(d)). Following the increasing stimulus intensity, the flicker responses were recorded more easily. Low frequencies such as 1 Hz, 2 Hz, and 3 Hz did not elicit flicker responses in the CSNB rats using dim light during dark adaptation.

## 4. Discussion

In the current paper, rod ERGs were not detected, and standard combined ERG waves were typically negative among the CSNB rats because of the *Cacna1f* mutation [7]. Cone ERGs were not detected in the RCD rats because the OPN1WN protein was absent, which affected the function of the middle wavelength-sensitive cone responses [12]. The ERG reflects the sum of the rod- and cone-mediated retinal responses and objectively evaluates visual function. We used temporal frequencies, dark and light adaptation states, and colored light to separate the cone and rod responses in the two retinal degeneration rat models.

## 5. Characteristics of Temporal Flicker ERGs in RCD Rats

Under scotopic conditions, the amplitude in the RCD rats elicited a small upward inflection point at 6 Hz. The amplitudes in the wild-type rats showed a linear decrease with increasing frequencies. Furthermore, the latencies in the RCD rats occurred early compared with the control rats at above 6 Hz (Figures 2(b) and 2(c)). A previous report showed a linear contrast response at the relatively low temporal frequency of 6 Hz because the rod-cone interaction influenced the temporal responses in the rats [26]. Our data demonstrates that more cancellations between the rod and cone responses may have occurred at 6 Hz in the normal rats. During dark adaptation, the first higher wave appeared at around 17 Hz to 18 Hz steadily in both the wild-type and the RCD rats (Figure 2(d)). The flicker response in the RCD rats was not detected at 20 Hz using white light or blue light (Figures 2(a) and 5(b)). The sensitivity to dim light of the rod system of the RCD rats was decreased at 17 Hz or 18 Hz and ended at 20 Hz. In contrast, the rod-driven CFFs of the wild-type rats were at 30 Hz. The results from the RCD and wild-type rats indicate that rods and cones influence flicker ERGs together from 17 Hz to 20 Hz. In addition, the responses rely on cone function from 20 Hz to 30 Hz under scotopic conditions. The cone-driven CFFs for the wild-type rat were sensitive to higher frequencies at about 62 Hz (Figure 3(a)) than in a previous report, which indicates that the rodents responds at about 50 Hz [27]. Armitage et al. [28] reported 51 Hz cone-driven CFFs and a different 21 Hz rod-driven CFF in the guinea pig retina. In humans, cone CFFs occur at about 60 Hz and rod CFF occur at 28 Hz [29, 30], and the rod CFF of mice occur at 26 Hz [31]. Our results for wild-type rats are similar to those of a previous report on humans. In this study, we eliminated the rod-cone interaction to determine the actual rod-driven CFF, which was at 20 Hz, and the cone-driven CFF was 62 Hz. During light adaptation, the RCD rats failed to produce any response, which is likely caused by damage to the cone functions of the RCD rats.

## 6. Characteristics of Temporal Flicker ERGs in CSNB Rats

Under scotopic conditions, the differences in amplitude decreased between the CSNB and wild-type rats with increasing frequency. For instance, the amplitudes for CSNB rats were only 1/10 those of the wild-type rats at 3 Hz, which increased to 1/7 at 5 Hz and 1/4 at 15 Hz (Figure 2(b)). Cones's function was primarily responsible for flicker responses in CSNB rats. Rods of rat are more sensitive to around 498 nm blue light, while cone of rat is sensitive to 505 nm and 370 nm [32]. In the present paper, we failed to record the blue flicker responses of the *Cacna1f *mutation rats using dim blue light. However, the temporal responses of the CSNB rats indicated sensitivity to higher temporal frequency and stronger luminance, which is characteristic of retinal cones. All of these results suggest that the rod pathway in CSNB rats was completely damaged, and the cones probably mainly contributed to flicker responses.

During light adaptation, the amplitudes of the CSNB rats significantly decreased, and the latencies were significantly delayed under a series of temporal frequencies compared with the wild-type rats. The function of cone system was also affected in the CSNB rats. The amplitude of cone flicker ERGs in the wild-type rats decreased at 10 Hz but increased in amplitude at 11 Hz (Figure 3(d)). In contrast, the temporal responses of the CSNB rats showed a more linear decline, which is similar to a previous report. Kondo injected monkeys with glutamate analogs to analyze the component origins of the flicker ERGs and found a decrease in amplitude at approximately 10 Hz. The responses were maximally 180° out of phase between the DBC and HBC pathways at about 10 Hz [17]. Although the mice did not exhibit a decrease in cone ERGs, the responses in the mouse cone ERGs were more linear than those of primates [19]. Thus, ERGs from different species have different characteristics. Three factors could be responsible for cone flicker responses: photoreceptors, DBCs, and HBCs [20]. The interaction between DBCs and HBCs is probably reduced in CSNB rats. One kind of cone path is blocked.

Kondo and Sieving indicted that the OFF-pathway contribute to responses of high frequencies at 32 Hz to 64 Hz in monkeys [17]. The rod- and cone-driven CFFs in the CSNB rats were both 25 Hz. Temporal responses at higher frequencies were absent in the CSNB rats. The results indicate that the survival of CSNB rat cones is probably related with the DBC pathway, which contributes to low frequencies in the cone system. However, we are uncertain whether HBC function was completely affected by the gene mutation. Analyzing the cells that survived in the cone system and the extent of its effect will be further studied in CSNB rats.

## 7. Interaction between Rod and Cone Systems

The temporal flicker responses under dark- and light-adapted conditions include rod-cone interactions [33, 34] and cone to DBC/HBC pathway communication [26, 35]. Rod-cone interference may manifest destructive interactions between the two separate system signals. One part of the interaction between the rod and cone pathways is generated by synaptic contacts between horizontal cells [36]. The CSNB rats lacked considerable horizontal cells and dendrites, although the number of photoreceptors was normal [7]. The signal transduction of rod systems horizontal cells must be reduced in CSNB rats, and the feedback of the light to cone was reduced as well. The cone flicker response in the CSNB rats must be relatively independent; therefore, the flicker ERGs in the CSNB rats were more linear than in the wild-type rats.

In human visible spectrum, cone is sensitive to 420 nm wavelength blue light, but the sensitiveness of blue light is 498 nm in rod. Rats have two types of cone-based vision [37], the two types are M/L sensitivecone and S sensitive cone (UV sensitive cone), whose wavelength is 505 nm and 370 nm, respectively [32]. And the rod is sensitive to around 480 nm wavelength blue light. We used blue filter (wavelength: 480 nm) covered the rats' eyes tight, then the dim light was gotten. So the cone of rat was not sensitive to the dim blue light we used. But the rod function was sensitive to 480 nm dim blue light. Removal of the restraint on rod response attenuated the rod-cone interaction in the RCD rats. This phenomenon could explain why the flicker ERGs in the RCD rats under scotopic conditions were bigger than those in the wild-type rats. Furthermore, the blue flicker amplitude was higher than that of the dim white light flicker ERGs (Figure 5(e)).

## 8. Conclusion

The cone-driven CFF of wild-type rats is around 62 Hz, which is higher than that previously reported. The rod-driven CFF in RCD rats ended at 20 Hz, and cone function disappeared. Both rod- and cone-driven CFFs of CSNB rats occur at 25 Hz. Rod function is completely damaged in CSNB rat, and the function of cone-DBCs pathway contributes to their visual function. Rod-cone interactions can be eliminated, and the actual sensitivity of rods is 20 Hz, as determined in RCD rats. The cone driven CFF is up to 62 Hz in albino rats. The separated flicker response and the rod/cone degeneration rat models provide more information regarding the characteristics of rods and cones in rats. The CSNB and RCD rats are two retinal degeneration models for studying the mechanism of ophthalmologic diseases.
